# From Blood to Brain: Adult-Born Neurons in the Crayfish Brain Are the Progeny of Cells Generated by the Immune System

**DOI:** 10.3389/fnins.2017.00662

**Published:** 2017-12-07

**Authors:** Barbara S. Beltz, Jeanne L. Benton

**Affiliations:** Neuroscience Program, Wellesley College, Wellesley, MA, United States

**Keywords:** serotonin, 5-HT, adult neurogenesis, immune system, dorsal giant neuron, neurogenic niche

## Abstract

New neurons continue to be born and integrated into the brains of adult decapod crustaceans. Evidence in crayfish indicates that the 1st-generation neural precursors that generate these adult-born neurons originate in the immune system and travel to the neurogenic niche via the circulatory system. These precursors are attracted to the niche, become integrated amongst niche cells, and undergo mitosis within a few days; both daughters of this division migrate away from the niche toward the brain clusters where they will divide again and differentiate into neurons. In the crustacean brain, the rate of neuronal production is highly sensitive to serotonin (5-hydroxytryptamine, 5-HT) levels. These effects are lineage-dependent, as serotonin's influence is limited to late 2nd-generation neural precursors and their progeny. Experiments indicate that serotonin regulates adult neurogenesis in the crustacean brain by multiple mechanisms: via direct effects of serotonin released from brain neurons into the hemolymph or by local release onto target cells, or by indirect influences via a serotonin-mediated release of agents from other regions, such as hormones from the sinus gland and cytokines from hematopoietic tissues. Evidence in crayfish also indicates that serotonin mediates the attraction of neural precursors generated by the immune system to the neurogenic niche. Thus, studies in the crustacean brain have revealed multiple roles for this monoamine in adult neurogenesis, and identified several pathways by which serotonin influences the generation of new neurons.

## Introduction

When the embryonic precursor cells die during late embryonic life, neural proliferation stops in most areas of the decapod crustacean brain (Harzsch, 2003; Sintoni et al., 2012). Exceptions to this are in the central olfactory and higher order processing pathways, where mitotic activity and the integration of new interneurons continue throughout life (Schmidt, 1997; Schmidt and Demuth, 1998; Harzsch et al., 1999; Schmidt and Harzch, 1999). The source of these adult-born neurons remained a mystery for many years, until the discovery of a neurogenic niche containing the 1st-generation neural precursors (Song et al., 2007; Sullivan et al., 2007a); these niche precursors generate a lineage of cells whose final progeny differentiate into neurons (Sullivan and Beltz, 2005b). However, the mystery deepened once again when it was discovered that the 1st-generation precursors do not self-renew (Benton et al., 2011). Calculations of cell-cycle time and counts of the niche precursor population showed that without self-renewal, the small pool of 1st-generation neural precursors should be rapidly depleted. But the niche is never exhausted and adult-born neurons continue to be produced throughout the long lives of these animals, leading to the conclusion that the neural precursors in the niche must be replenished from a source elsewhere in the animal. This paper reviews the intense hunt for the source of these cells, which ultimately led to the immune system and the identification of a specific type of circulating blood cell (hemocyte) that is attracted to the neurogenic niche, where these go through their first division as neural precursors; their daughters migrate along streams arising from the niche, finally arriving at brain cell clusters containing interneurons in the olfactory pathway. Ultimately, these cells undergo at least two more divisions over 1–2 weeks, before the progeny differentiate into neurons.

Serotonin has long been known to regulate neuronal proliferation in the embryonic and adult brains of crustaceans (Benton et al., 1997, 2008), as in vertebrate brains (Brezun and Daszuta, 1999, 2000; Banasr et al., 2004; Lledo et al., 2006). In crayfish, serotonin is involved in multiple functions contributing to adult neurogenesis, including the attraction of immune cells to the niche (Benton et al., 2011) and the timing of cell divisions in the neural precursor lineage (Zhang et al., 2011). One major source of serotonin in the brains of crustaceans is the dorsal giant neuron (DGN). Tests in which the DGN was electrically stimulated have shown that serotonin released from this neuron directly alters the rate of adult neurogenesis in the crayfish brain. Further, by activating a cytokine pathway, serotonin is emerging as a critical link between the immune and nervous systems.

## Adult neurogenesis in the decapod crustacean brain

### The neural precursor lineage in the adult brain has been identified

**Figure 1:**
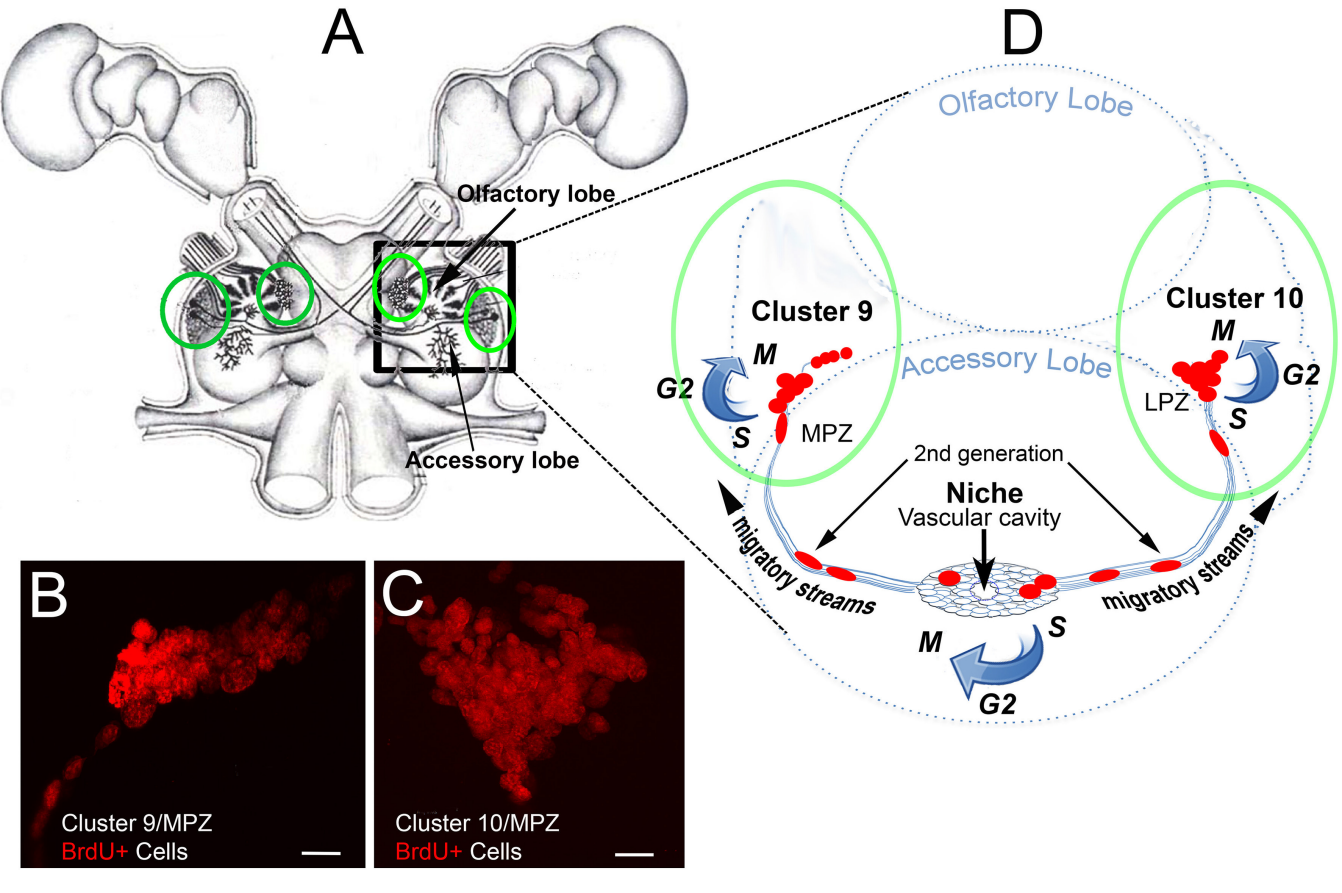
The cellular machinery producing adult-born neurons in the crayfish brain**. (A)** Drawing of the eureptantian (crayfish/lobster) brain including the eyes and optic ganglia of the lateral protocerebrum, and showing the locations of the cell body clusters and neuropils in the brain (supraesophageal ganglion). Bilateral soma clusters 9 and 10 (circled in green, **(A)** are locations where neurogenesis persists in the adult brain, shown with bromodeoxyuridine labeling (red) of S-phase cells in **(B,C)**. These cell clusters lie beside two neuropil regions in the deutocerebrum, the olfactory (OL) and accessory (AL) lobes, shown diagrammatically in **(D)** (right hemibrain). The neurogenic niche lies on the ventral surface of the accessory lobes, just beneath the membrane ensheathing the brain. The niche contains bipolar cells that have short processes that project to the rim of a vascular cavity, and long processes that form migratory streams extending to Clusters 9 or 10, along which the daughters of 1st-generation neural precursors in the niche migrate. Scale bars: **(A,B)**, 20 μm.

Most neurons in the decapod brain are born during embryonic development and are the descendants of large precursor cells, the neuroblasts (for review see Harzsch, 2003). Neuroblasts are active during embryonic development, dividing asymmetrically, generating specific neural lineages before dying during the period around hatching (Sintoni et al., 2012). Therefore, neural proliferation in most regions of the decapod brain ends during late embryonic or early postembryonic development. However, the cell cycle resumes after hatching in the local (Cluster 9) and projection (Cluster 10) neuron clusters located in the midbrain (deutocerebrum) (Figure 1; Harzsch et al., 1999; Schmidt and Harzch, 1999); the production of these adult-born neurons and the roles of serotonin in this process will be the focus of this review. Neurons in these clusters innervate the primary olfactory processing areas (olfactory lobes; OLs) and higher-order processing areas (accessory lobes; ALs) that integrate olfactory, visual and mechanosensory information. Life-long neurogenesis is also found in the clusters of olfactory sensory neurons in the antennules (Steullet et al., 2000; Sullivan and Beltz, 2005a; Tadesse et al., 2011) and among neurons in the visual pathway (Sullivan and Beltz, 2005b).

**Figure 2:**
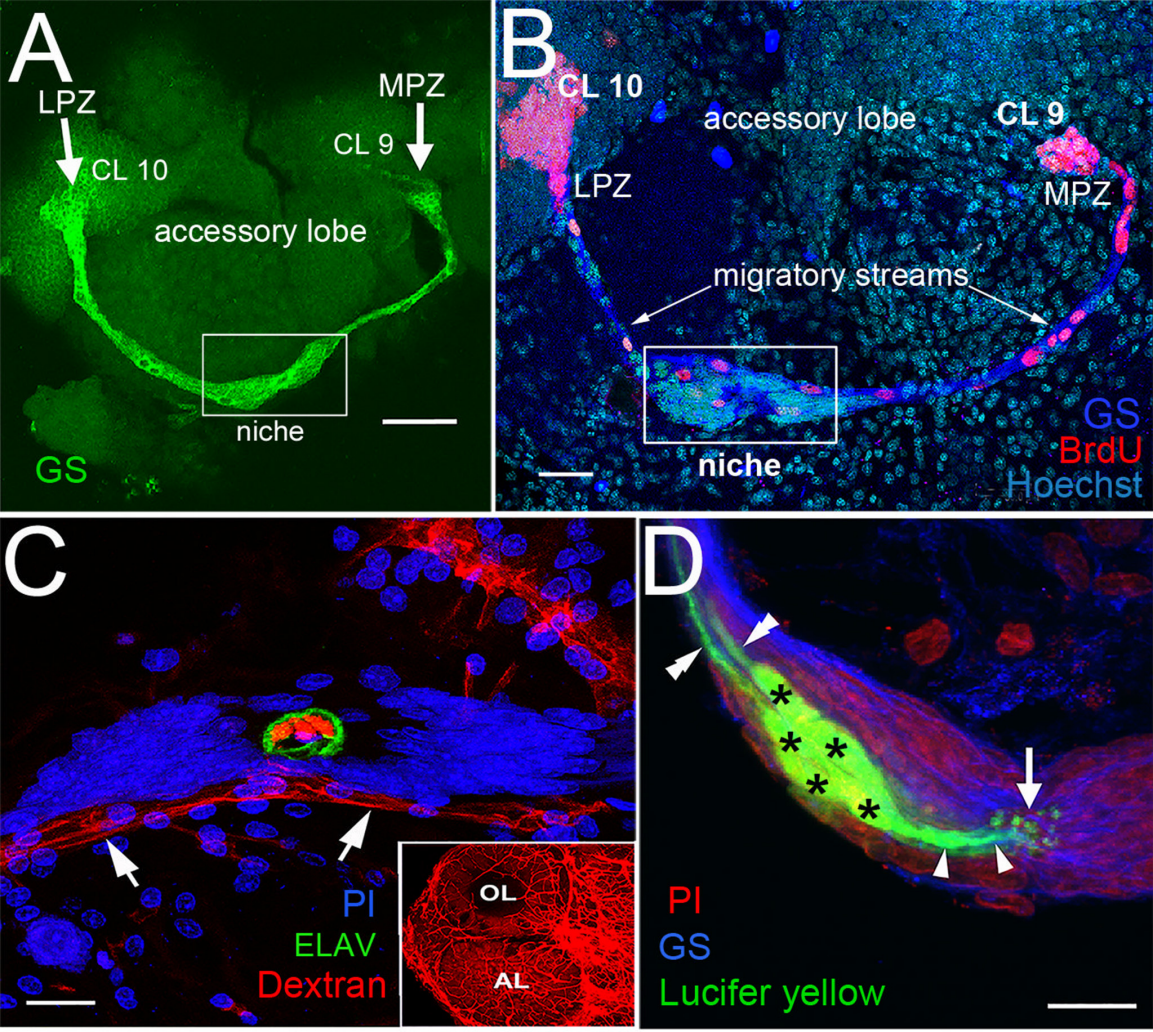
The proliferative system maintaining adult neurogenesis in the crayfish (*Procambarus clarkii*) brain. **(A)** The lateral (LPZ) and medial (MPZ) proliferation zones are contacted by the processes of a population of cells immunoreactive to GS (green) whose somata are located in a neurogenic niche (white box) on the ventral surface of the brain. **(B)** Left side of the brain of *P. clarkii* labeled immunocytochemically for the S-phase marker BrdU (red). Labeled cells are found in the LPZ contiguous with Cluster 10 (CL 10) and in the MPZ near Cluster 9 (CL 9). The two zones are linked by a chain of labeled cells in a migratory stream that originates in the boxed region labeled “niche.” Labeling for glutamine synthetase (blue), BrdU (red), and Hoechst (cyan) is shown. **(C)** The vascular connection to the cavity in the center of the niche was demonstrated by injecting dextran tetramethylrhodamine dye into the cerebral artery. The cavity, outlined by its reactivity to an antibody to *ELAV* (green), contains the dextran dye (red), which is also contained within a larger blood vessel that lies beneath the niche (arrows). PI (blue) labeling of the niche cell nuclei is also shown. Inset: dextran-filled vasculature in the olfactory (OL) and accessory (AL) lobes on the left side of the brain. **(D)** Niche cells (green), labeled by intracellular injection of Lucifer yellow, have short processes (arrowheads) projecting to the vascular cavity (arrow) and longer fibers (double arrowheads) that fasciculate to form the tracts projecting to the LPZ and MPZ, along which the daughters of the niche cells (2nd-generation neural precursors) migrate. Glutamine synthetase (GS), blue; propidium iodide (PI), red. Scale bars: **(A)**, 100 μm; **(B)**, 30 μm; **(C,D)**, 20 μm (A, C and D from Sullivan et al., 2007a).

The production of new midbrain neurons has been demonstrated in sexually mature adults in several decapod groups (Schmidt and Demuth, 1998; Schmidt and Harzch, 1999), suggesting that adult neurogenesis is a general feature of the decapod brain. However, evidence for the differentiation of newly born cells into neurons expressing the appropriate transmitters has been obtained only for crayfish (Astacida; Sullivan and Beltz, 2005b) and spiny lobsters (Achelata; Schmidt, 2001). It has been proposed that the neural precursors supporting adult neurogenesis in the spiny lobster *Panulirus argus* may be self-renewing neuroblasts that survived after embryonic life (Schmidt and Derby, 2011), but direct tests of this hypothesis have not yet been conducted. However, the precursor cell lineage producing adult-born neurons in the midbrain of the crayfish *Procambarus clarkii* has been identified (Figure 1). The 1st-generation neural precursors are located in two neurogenic niches lying on the ventral surface of the brain, just beneath the sheath (Song et al., 2007; Sullivan et al., 2007a). The niche cells are immunoreactive for glutamine synthetase (GS), an enzyme that converts glutamate to glutamine, and which is also a marker of astrocytes (Anlauf and Derouiche, 2013) and radial glial-like cells including neural stem cells in the CNS of fish (Wen et al., 2008, 2009). When the crayfish brain and niche are labeled with antibodies for GS, the neurogenic cells in the brains of adult crayfish are revealed (Figures 2A,B; Sullivan et al., 2007a). As in mammals, the neurogenic niches supporting adult neurogenesis in the crayfish brain are intimately associated with the vasculature, as these lie on blood vessels that communicate with the niche via a vascular connection (Figure 2C) (Sullivan et al., 2007a; Chaves da Silva et al., 2012). This vascular cavity contains amorphous non-cellular material that reacts with alcian blue and periodic acid-Schiff (Bazin, 1969), suggesting a glycidic substance (Chaves da Silva et al., 2012).

The vast majority of cells in the niche are bipolar, with long processes that project from the niche to either Cluster 9 or 10 and short processes that terminate at the vascular cavity (Figure 2D). When the 1st-generation neural precursors in the niche divide, their daughters (2nd-generation neural precursors) migrate along these processes to Cluster 9 or 10, forming streams that deliver the niche descendants to the brain cell clusters. Thus, niche cells in the crustacean brain appear to function as precursor and support cells; it is now thought that these may represent two distinct cell types. The 2nd-generation neural precursor cells require 5–7 days to migrate along the streams (Benton et al., 2011). After reaching the proliferation zones in Cluster 9 or 10, they divide again, their progeny differentiating into interneurons innervating the olfactory and/or accessory lobes (Sullivan and Beltz, 2005b; Sullivan et al., 2007a,b). A wave of cell death culls the newborn cells during the first 2 weeks after birth, but by 4 weeks the surviving cells have begun to express neurotransmitters that are typical of local (Cluster 9) and projection (Cluster 10) neurons in the olfactory pathway (Kim et al., 2014).

Many of the events underlying the production of neurons in the adult crayfish brain are reminiscent of adult neurogenesis in the mammalian brain, suggesting that these processes may be grounded in common ancestral mechanisms that have been retained in a phylogenetically broad group of species, or, alternatively, that reflect convergence on common mechanisms (Sullivan et al., 2007a). These similarities include the presence of vascularized niches that house the neural precursors and the directed migration of their descendants, conservation in molecular pathways underlying neuronal differentiation, and extensive parallels in the environmental and endogenous factors that regulate adult neurogenesis (Beltz et al., 2011; Brenneis et al., 2017). However, there also are important differences that distinguish adult neurogenesis in crayfish. For example, in contrast to the coexistence of several precursor cell generations in the mammalian neurogenic niche, the generations of cells in the neural precursor lineage in crayfish are compartmentalized: the 1st -generation precursors in the crayfish niche are separated from their progeny in the migratory streams (2nd-generation precursors), which in turn are segregated from their descendants in the proliferation zones associated with Clusters 9 and 10 (3rd-generation and later precursors). As a result, the lineage relationships among the precursors are clear and changes in the numbers of cells in each generation are easily assessed (e.g., Zhang et al., 2011).

### First-generation neural precursors in the crayfish brain are not self-renewing

**Figure 3:**
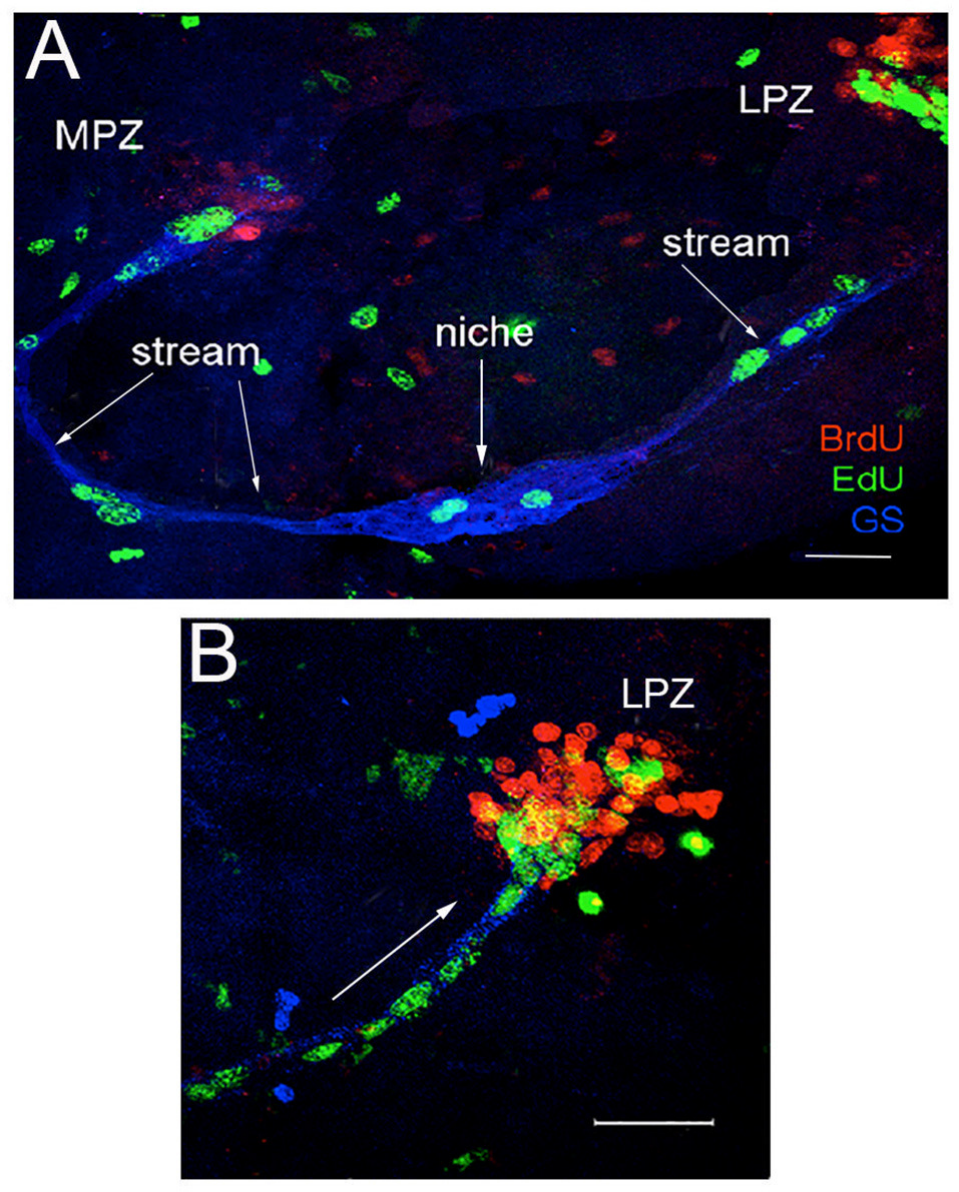
Double-nucleoside labeling demonstrates that the niche cells are not self-renewing. Crayfish were incubated for 24 h in BrdU (red), then maintained in pond water for 7 days. Six hours before sacrifice, they were treated with EdU (green). Brains also were labeled immunocytochemically for glutamine synthetase (blue) to reveal the niche and streams. **(A)** BrdU+ cells were found only in the proliferation zones (MPZ and LPZ), not in the niche. Only EdU labels S-phase cells in the niche, showing that niche cells do not retain BrdU, and thus that divisions are not self-renewing. These labeling patterns also demonstrate that migration is unidirectional, away from the niche. **(B)** Higher magnification image of the LPZ. Arrow in B indicates the direction of neural precursor cell migration. Scale bars: **(A)**, 100 μm; **(B)**, 50 μm. Adapted from Benton et al. (2014).

Unlike embryonic neural stem cells (neuroblasts) in crustaceans, the 1st-generation precursors that support adult neurogenesis undergo morphologically symmetrical divisions (Zhang et al., 2009). Pulse-chase double-nucleoside labeling allows the separate detection of two different nucleosides (5-bromo-2′-deoxyuridine [BrdU] and 5-ethynyl-2′-deoxyuridine [EdU]), which are incorporated into cells that are synthesizing DNA during S phase of the cell cycle. BrdU (presented first) is not retained in the 1st-generation precursors, and is replaced with EdU (presented 3-7 days later) in niche cells (Figure 3). Rapid division and dilution of the BrdU label cannot explain the absence of nucleoside retention by the precursor cells in the niche, because the cell cycle of the 1st-generation precursors is relatively long [~48 h; (Benton et al., 2011)]. Thus, because the 1st-generation neural precursors in the crayfish neurogenic niche do not retain BrdU or EdU, we have concluded that these are not self-renewing and that both daughters of a niche cell division enter the streams; this is supported by the observation of pairs of labeled cells migrating together in the streams proximal to the niche. However, in spite of this apparent lack of self-renewal capacity among the 1st-generation neural precursors, these cells are never depleted and neurons continue to be generated throughout the long lives of these animals. It therefore follows that cells generated elsewhere in the organism must be replenishing the pool of 1st-generation neural precursors in the niche (Zhang et al., 2009).

**Figure 4:**
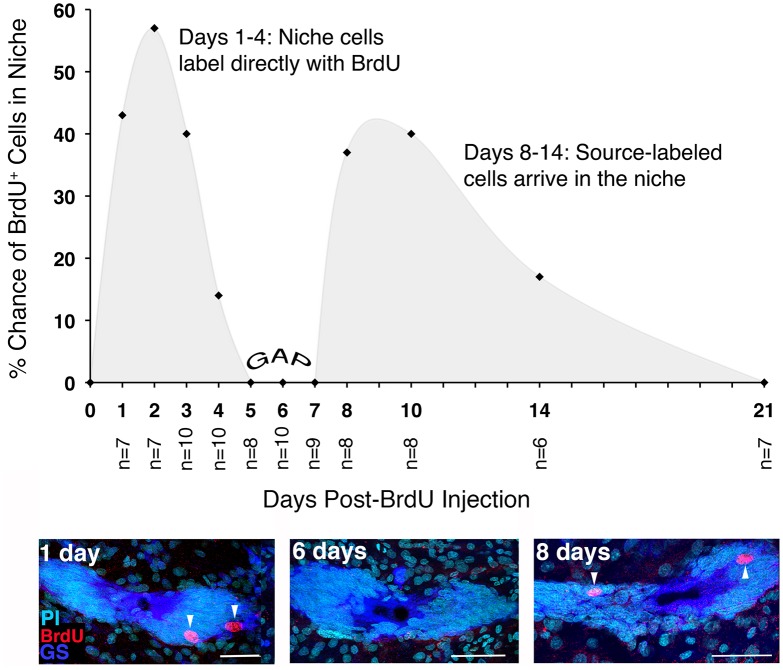
Actively proliferating (BrdU+) cells in the neurogenic niche have a bimodal temporal distribution. BrdU+ cells were quantified in the niches of crayfish that were sacrificed daily for 1 week after BrdU injection, and at intervals thereafter for 21 days. The probability of observing BrdU+ cells in the niche was then plotted for each of the sampling days. BrdU+ cells were observed in the niche on days 1–4 following injection (shown in the day 1 image below graph). On days 5–7, the niches contained no BrdU+ cells (GAP). However, between days 8 and 14 after injection, BrdU+ cells were once again observed in the niche. Adapted from Benton et al. (2014). Scale bar: 50 μm.

The existence of an extrinsic source of neural precursors was confirmed by experiments in which groups of adult crayfish were injected once with BrdU; nucleoside labeling of 1st-generation precursors in the niche was then documented daily for 1 week and at intervals until 21 days after injection. We anticipated that 1st-generation precursors in the niche that are in S phase during the BrdU exposure would label with BrdU, as would proliferating cells in other tissues, including those that produce neural precursors. However, we hypothesized that neural precursor cells from extrinsic sources would require time after BrdU incorporation to complete their lineages and travel to the niche. Therefore, we expected that cells from an external source would arrive at the niche after a delay. Indeed, BrdU labeling among 1st-generation neural precursors in the niche was observed reliably on days 1–4 following exposure (Figure 4). No BrdU+ cells were found in the niche on days 5–7 following BrdU exposure. We attributed this “gap” in labeling to the fact that BrdU has a ~2 day clearing time in crayfish (Benton et al., 2011), and thus after this period BrdU would no longer be available. Further, cells instrinsic to the niche that had incorporated BrdU during the initial exposure would have divided and migrated into the streams, as these cells have an 18–24 h cell cycle time (Benton et al., 2011). However, on days 8–14 following BrdU injection, intensely labeled cells were once again observed in the niche. As BrdU was no longer available, we concluded that this delayed labeling of niche cells must be due to cells that traveled to the niche that had incorporated BrdU while in their source tissue (Benton et al., 2014). These data also suggest a “just-in-time” replenishment in which neural precursors arrive at the niche and rapidly divide; their daughters then migrate to brain clusters 9 and 10. This experiment therefore supports the existence of an extrinsic source that replenishes neural precursors in the niche, and also indicates that the non-mitotic cells in the niche may be a distinct cell type that serves a supporting role. Consistent with this interpretation, four morphologically distinct niche cell types have been described, with Type 1 cells by far the most numerous. These bipolar cells have long processes that extend to either Cluster 9 or 10, forming the streams along which the 2nd-generation neural precursors migrate. They also have short processes with microvillar extensions that contact the vascular cavity, and junctional complexes between adjacent cells; these features suggest a role in regulating transport and communication between the vasculature and the niche cells (Chaves da Silva et al., 2012). Beyond their morphological characteristics, we know very little about the other three niche cell types.

Identifying the source(s) of the 1st-generation neural precursors has been the priority in our recent work. *In vitro* studies were conducted in which cells were isolated from several different types of tissues. These were labeled with CellTrackerTM Green CMFDA (CTG; Invitrogen), a fluorescent marker, and were then incubated at 18°C with freshly dissected, desheathed crayfish brains and their associated neurogenic niches. After 6 h, the distribution of labeled cells in each culture dish was recorded. Most cell types were distributed evenly in the culture dishes and showed no affinity for the brains or niches (e.g., cells extracted from green gland, hepatopancreas and hematopoietic tissues). In contrast, hemocytes (blood cells) expressed a strong affinity for the niche; these were observed in the vascular cavity or among the niche cells in 77% of cultures. In addition, many of the CTG-labeled cells were immunoreactive for glutamine synthetase, which is a marker of all niche cells in *P. clarkii*. These *in vitro* studies thus provided the first direct evidence that the immune system might be the source of neural precursor cells.

### The immune system generates neural precursors supporting adult neurogenesis

The next goal of our work was to explore the relationship between adult neurogenesis and the immune system, and to test the *in vivo* competence of hemocytes as precursors of adult-born neurons in the crustacean brain. Invertebrates do not generate adaptive immune responses, as they do not have oxygen-carrying erythrocytes or blood cells of the lymphoid lineage. However, these organisms do have a sophisticated innate immune system in which hemocytes play a dominant role, participating in both innate immunity and blood clotting (Lin and Söderhäll, 2011). Freshwater crayfish contain discrete hematopoietic tissues (Noonin et al., 2012; Chaves da Silva et al., 2013) that generate three morphologically distinct types of circulating hemocytes (hyaline, semi-granular and granular cells; Chaga et al., 1995) throughout an animal's long lifetime (up to 20 years in some species). Hemocytes are synthesized and partly differentiated through two main cell lineage pathways in the hematopoietic tissues, but the final development into functional hemocytes takes place after release into the circulation (Söderhäll et al., 2003; Wu et al., 2008). The crustacean immune system also generates prokineticin-family cytokines; these “astakines” promote the proliferation and release of hemocytes from hematopoietic tissues (Söderhäll et al., 2005; Lin et al., 2010). In vertebrate species, prokineticins play roles in circadian regulation, angiogenesis and neurogenesis; adult neurogenesis in the olfactory bulb requires prokineticin 2 (Ng et al., 2005).

**Figure 5:**
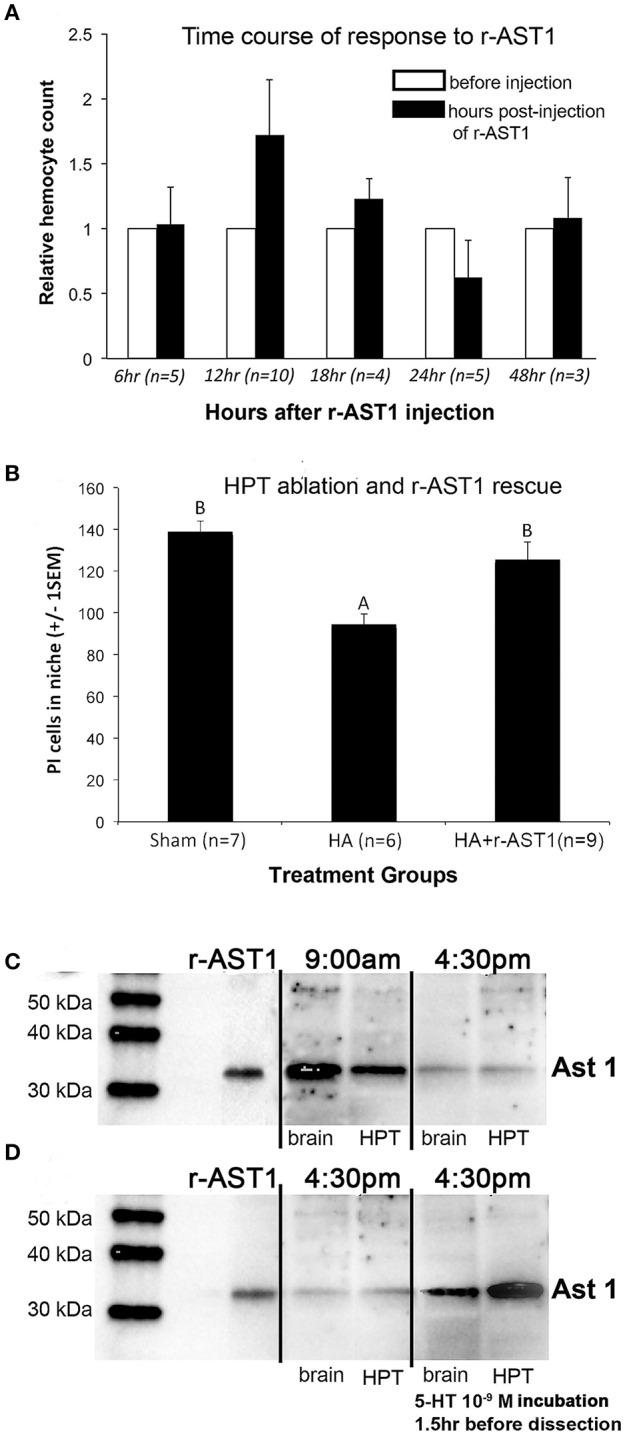
Astakine 1: Effects on hemocyte release and the neurogenic niche, and regulation by time of day and serotonin. **(A)** Time course of release of hemocytes after r-AST1 injection into *P. clarkii*. The relative hemocyte count was calculated as the total hemocyte count (THC) at each time point divided by THC prior to r-AST1 injection. The greatest release of hemocytes occurs 12 h after r-AST1 injection (*p* = 0.0002). **(B)** Crayfish in which hematopoietic tissues were partially ablated (HA) have a significantly lower total number of niche cells (propidium iodide [PI]-labeled) compared to shams (ANOVA, *F* = 2.19, *p* < 0.002). Injection of r-AST1 into HA crayfish 48 h prior to sacrifice (HA+r-AST1) restores the niche cell counts to sham values. **(C)** Western blot of *P. clarkii* AST1 and *P. leniusculus* r-AST1 at 9 a.m. and 4:30 p.m., showing that AST1 expression varies depending on time of day, with higher expression in the morning as previously reported for *P. leniusculus* (Watthanasurorot et al., 2011). **(D)** After serotonin (10^−9^ M) incubation, the 4:30 p.m. expression of AST1 increases. A, B published in Benton et al. (2014).

Our first experiments exploring the relationship between the immune system and adult neurogenesis in crayfish assessed whether total hemocyte counts (THC) were correlated with the number of cells in the neurogenic niche. THC was manipulated either by ablating part of the hematopoietic tissue to reduce THC, or by injecting the crustacean cytokine astakine 1 (AST1) into crayfish to increase THC. AST1 selectively promotes the proliferation and release of semi-granular hemocytes in the crayfish *P. leniusculus* (Lin et al., 2010) and in *P. clarkii* (Benton et al., 2014, 2017). The THC manipulation studies demonstrated a close relationship between the system producing adult-born neurons in the brain and the innate immune system of *P. clarkii*. First, the immune system regulates the neural precursor lineage by releasing AST1, which promotes hemocyte release from hematopoietic tissues (Figure 5A), thus raising THC. Second, THC and the number of cells in the neurogenic niche are positively correlated; manipulating the levels of circulating hemocytes results in highly predictable changes in the niche, with AST1 raising and hematopoietic tissue ablations reducing niche cell numbers (and THC). Further, the reduced niche cell numbers that result after partial hematopoietic tissue ablation can be rescued by injecting recombinant AST1 (r-AST1) prior to sacrifice (Figure 5B; Benton et al., 2014). Finally, r-AST1 increases the number of BrdU-labeled cells in the niche and streams, suggesting an effect on cell cycle time. These results demonstrate that the immune system regulates the neurogenic niche dynamically and implicates semi-granular hemocytes in this process, because AST1 specifically regulates the release of this blood cell type and also is capable of rescuing the reduction in niche cell counts after hematopoietic tissue ablations.

**Figure 6:**
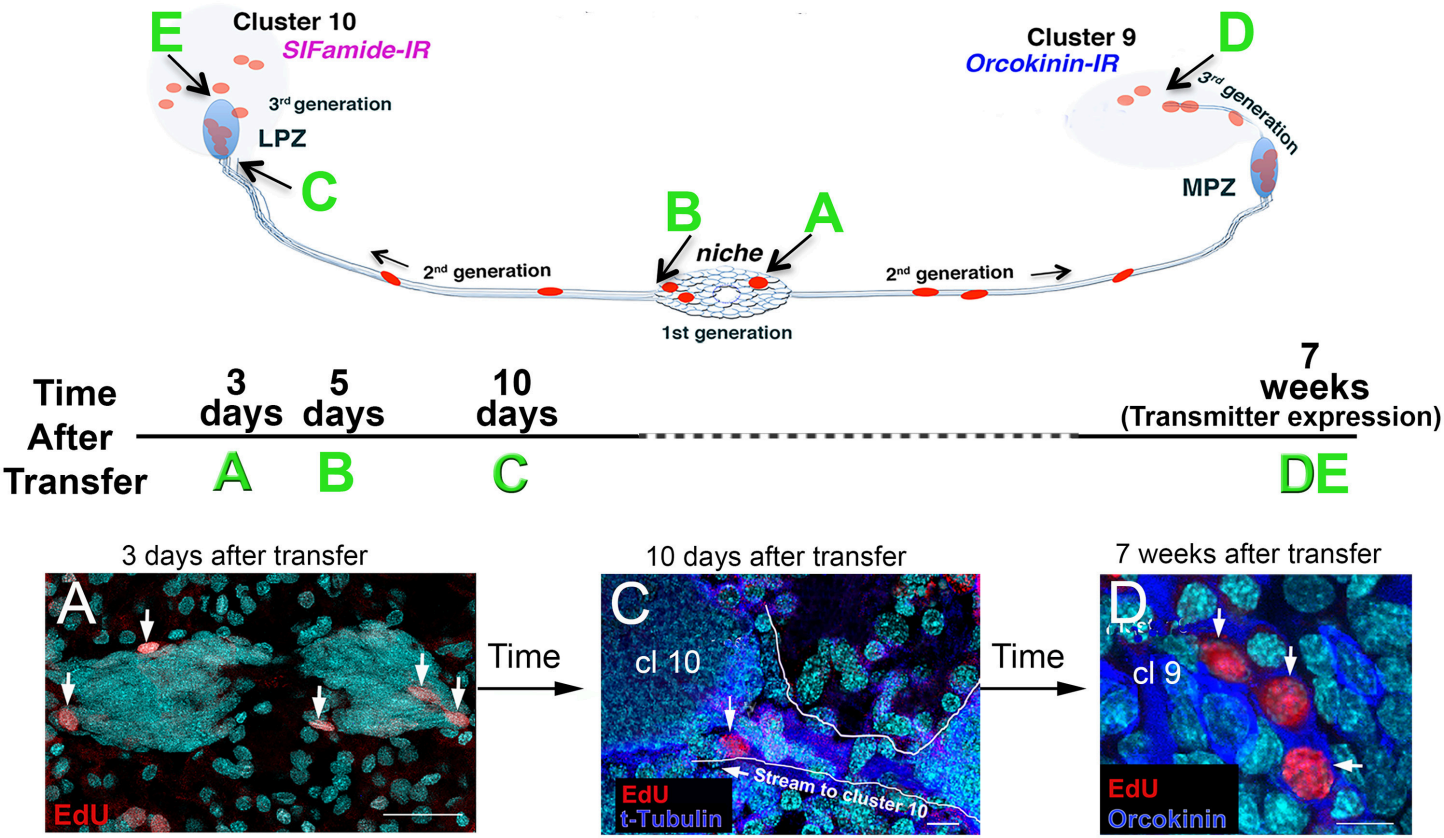
EdU+ cells were observed in the niche, streams, and in Clusters 9 and 10 of recipient crayfish after adoptive transfer of EdU+ hemocytes from donor animals. The schematic diagrams illustrate (top) the locations of the EdU+ cells that were observed following adoptive transfers, and (middle) the experimental timeline for each sample (time after transfer, **A–E**), and (bottom) images from *P. clarkii* **(A,D)** and *P. leniusculus* **(C)**. All samples in these images **(A,C,D)** were labeled with the nuclear marker Hoechst 33342 (cyan). **(A)** Three days after adoptive transfer of hemocytes, EdU+ cells (red) were observed in the niche. **(C)** Ten days after hemocyte transfer, cells were observed in the distal ends of the streams, near Clusters 9 and 10 (arrow). Immunoreactivity for tyrosinated tubulin (blue) highlights the migratory stream, which is also outlined in white. **(D)** Seven weeks after adoptive transfer of hemocytes from donor to recipient crayfish, EdU+ cells (red) were observed in cell Clusters 9 and 10. Some of these cells in Cluster 9 express orcokinin, a peptide transmitter used by many Cluster 9 cells. Examples of cell labeling at B (5 days, in the niche) and at E (7 weeks, in Cluster 10) are not shown (but see Benton et al., 2014). Scale bars: **(A)**, 40 μm; **(C)**, 10 μm; **(D)**, 10 μm. Adapted from Benton et al. (2014).

Adoptive transfer methods were then used to ask whether hemocytes labeled with EdU in donor crayfish would be attracted to the niche in recipient crayfish, as they were *in vitro*. Despite the fact that labeled donor cells represented less than 1% of circulating hemocytes in the recipients, EdU+ cells were nevertheless found in the neurogenic niches of recipient crayfish within a few days of transfer (Figure 6; Benton et al., 2014). Over a period of several days following hemocyte transfer, EdU+ cells were observed in both the migratory streams and in Clusters 9 and 10 in the brain, where adult-born neurons normally differentiate. And, by 7 weeks following hemocyte transfers, EdU+ cells in these cell clusters expressed transmitter substances typical of cells in these regions (Figure 6). These studies therefore showed that cells circulating in the hemolymph can become neural precursors when transferred directly to recipient crayfish (Benton et al., 2014). Additional adoptive transfer experiments in which specific hemocyte types were tested have shown that semi-granular hemocytes are the only circulating cell type that is attracted to the niche, strongly implicating these as the cells that replenish the pool of neural precursors in the niche (Cockey et al., 2015; Benton et al., 2017).

Labeled cells harvested from other tissues that are adoptively transferred using the same methods do not result in labeling of cells in the niche, migratory streams or brain cell clusters, suggesting that the attraction of hemocytes to the neurogenic niche is specific and not a general property of all transferred cell types. In addition, transferred hemocytes are not incorporated into other rapidly proliferating tissues such as hepatopancreas or hematopoietic tissue, also indicating that the interaction of donor hemocytes with the niche results from a selective affinity between these tissues. Our experiments therefore suggest that circulating blood cells, specifically the semi-granular hemocytes, are able to become neural precursors, and that these cells and their descendants successfully navigate the many challenges involved in attraction to the niche, migration along the streams, neural differentiation and survival. Further, the generation of neural precursors by the innate immune system challenges the canonical view that ectodermal tissues are the sole source of neurons in the brain.

## Serotonin regulates adult neurogenesis

The generation of adult-born neurons in the decapod crustacean brain is highly regulated. Modulatory factors include the living conditions (i.e., enriched vs. deprived environments) (Sandeman and Sandeman, 2000; Ayub et al., 2011), hormonal cycles (Harrison et al., 2001), diet (Beltz et al., 2007), seasonality (Hansen and Schmidt, 2001), the day/night cycle (Goergen et al., 2002), nitric oxide (Benton et al., 2007) and serotonin (Benton et al., 2008; Zhang et al., 2011). As described below, serotonin works at multiple levels to influence neurogenesis: by regulating the expression of the cytokine astakine, which in turn promotes the differentiation and release of semi-granular hemocytes from hematopoietic tissues; as a mediator of hemocyte attraction to the neurogenic niche; and as a cell cycle regulator acting directly on specific neural precursor generations. However, as is clear from the variety of environmental and intrinsic factors that influence the generation of new neurons, the regulation of adult neurogenesis is a complex process involving actions of multiple modulators working in concert. Therefore, although serotonin's roles are highlighted here, these influences will be integrated in the living organism with the actions of the many other regulatory agents in play at a given time.

The adult-born neurons in Clusters 9 and 10 project to the olfactory and accessory lobes, which receive a dense serotonergic innervation, much of which is contributed by the dorsal giant neurons (DGNs). Experiments in embryonic lobsters demonstrated that when serotonin is depleted, the growth and maturation of the olfactory and accessory lobes are selectively delayed compared to these regions in the brains of embryos with normal serotonin levels (Benton et al., 1997). Subsequent studies demonstrated that this retarded development is due to a failure of Cluster 9 and 10 neurons to branch and grow into the olfactory and accessory lobes (Sullivan et al., 2000), and to reduced proliferation and survival of neurons in both cell clusters (Beltz et al., 2001; Benton and Beltz, 2001). Thus, serotonin influences both neurogenesis and neuronal differentiation in the embryonic brain.

### Regulation of adult neurogenesis by serotonin

Serotonin also is a potent regulator of neurogenesis in the adult crustacean brain, acting directly to increase proliferation in the neurogenic lineage at low levels (10^−9^–10^−10^ M), or indirectly suppressing neurogenesis by a serotonin-mediated release of hormones from the sinus gland, which requires higher concentrations (10^−4^ M) of the monoamine (Benton et al., 2008, 2011). The neurogenic niche does not appear to receive neural innervation, although serotonergic fibers have been reported in the proliferation zone of Cluster 10 (Beltz et al., 2001). In addition, both the niche and BrdU-labeled cells in the proliferation zone of Cluster 10 lie close to or directly adjacent to blood vessels (Sullivan et al., 2007a; Sandeman et al., 2009). Together, these features suggest that the serotonergic stimulation of neurogenesis may be mediated by serotonin that is circulating at low levels in the hemolymph.

**Figure 7:**
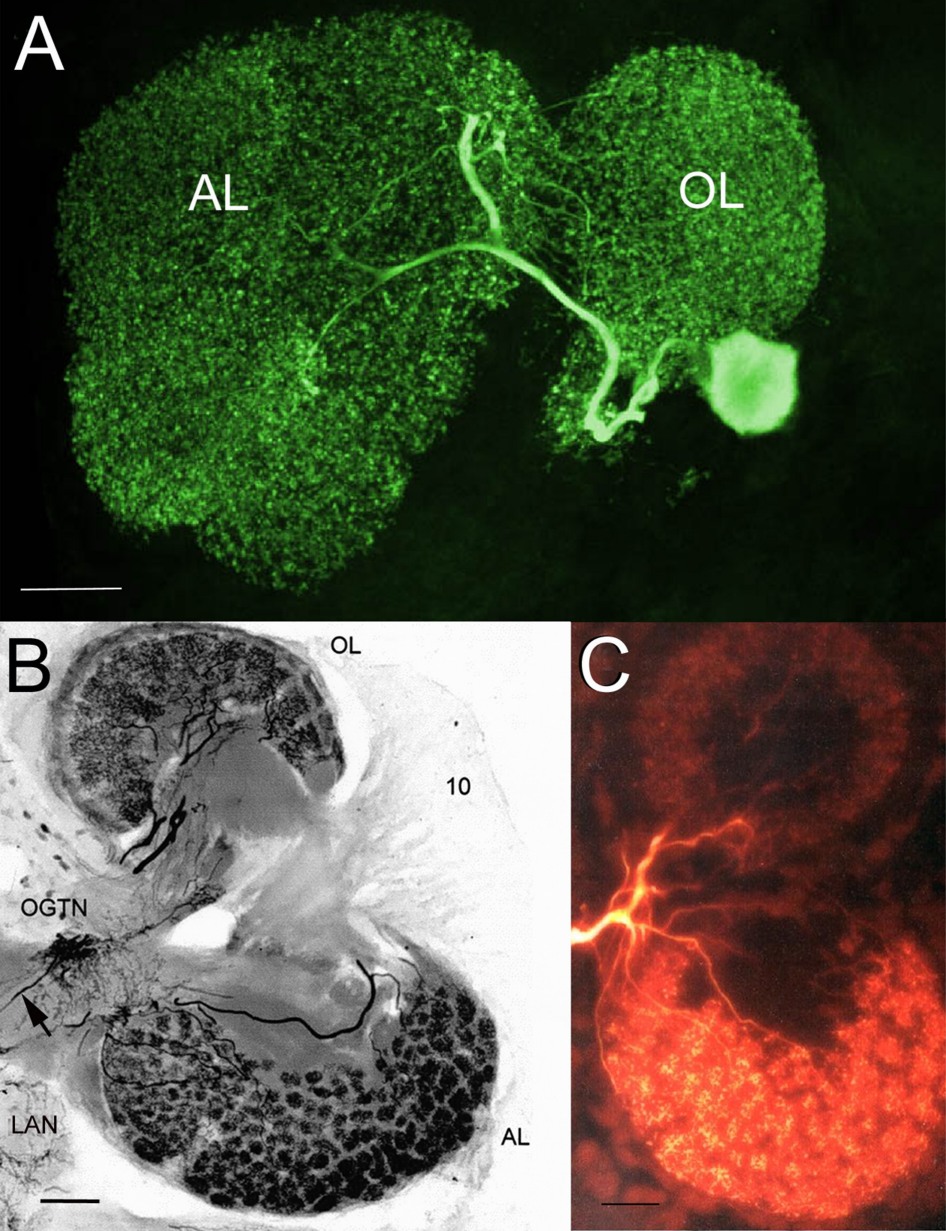
The serotonergic dorsal giant neuron (DGN) innervates each and every glomerulus in the olfactory and accessory lobes. **(A)** Stacked confocal image of a neurobiotin-filled DGN in a lobster (*Homarus americanus*) embryo, revealing the massive projections of the DGN within the olfactory and accessory lobes. **(B)** Photomicrograph of a thick section (100 μm) of the right side of the adult brain of the Australian crayfish *Cherax destructor* immunolabeled for serotonin. The DGN is the only serotonergic neuron that projects to the AL, but shares its projection into the OL with at least two other serotonergic midbrain neurons. The large cell body of the DGN is dorsal and out of the section plane, but the thin primary neurite that connects it with the olfactory globular tract neuropil (OGTN) before it projects into the OL and AL is observed (arrow). 10, Cluster 10 cell bodies; LAN, lateral antennular neuropil. **(C)** Neurobiotin injection of the DGN in an adult *C. destructor* brain illustrating the projections of this neuron to virtually all glomeruli in the OL and AL. Scale bars: **(A)**, 50 μm; **(B,C)**, 100 μm. Adapted from Beltz and Sandeman (2003).

While there may be multiple origins of circulating serotonin, one major source is the DGNs. These very large neurons have massive projections that infiltrate each and every glomerulus in the olfactory and accessory lobes (Figure 7; Sandeman and Sandeman, 1994; Sandeman D. et al., 1995; Sandeman R. E. et al., 1995). These structural characteristics suggest that the DGNs are sensory integrators, with the potential to receive olfactory, visual and mechanosensory information, and to influence processing in these regions. In addition, these giant neurons also release serotonin into the hemolymph (Sandeman et al., 2009). We therefore tested the hypothesis that the serotonergic DGN regulates adult neurogenesis by examining the influence of DGN stimulation on BrdU incorporation into cells in the proliferation zone of Cluster 10, an area known to be sensitive to serotonin levels (Benton et al., 2008), and where the final divisions of neural precursors take place prior to differentiation into projection neurons.

Adult neurogenesis continues in dissected, perfused crayfish (*Cherax destructor*) brain preparations, although at a slower rate than *in vivo*; however, 10^−9^ M serotonin added to the perfusate bathing the brain restores the rate of neurogenesis to *in vivo* levels (Sandeman et al., 2009). Therefore, a dissected brain preparation was used to depolarize or hyperpolarize the serotonergic DGN on one side of the brain, while using the contralateral unstimulated side as the paired control; after a 6-h stimulation period and perfusion of the brain with BrdU, brains were fixed and the numbers of BrdU-labeled cells in Cluster 10 on each side of the brain were counted. Stimulation and the generation of action potentials in the DGN on one side of the brain was correlated with an increase in BrdU incorporation into cells in Cluster 10 on that side compared to the unstimulated side. Hyperpolarization of the DGN, on the other hand, was associated with a small decrease in the number of BrdU-labeled cells on the stimulated relative to the control side of the brain. High-performance liquid chromatography (HPLC) was used to measure serotonin levels in the perfusate of stimulated brains; serotonin levels increase roughly10-fold, confirming that serotonin is released when the DGN is active. Further, perfusate levels of serotonin collected during stimulation ranged from 10 ^−11^–10 ^−13^ M; factoring in the added dilution in the perfusate following serotonin release implies values at the sites of neural proliferation that are optimal for increasing the rate of neurogenesis (10 ^−9^–10 ^−10^ M) (Benton et al., 2008). These data suggest that suprathreshold excitation of the DGN results in a non-synaptic release of serotonin into the vascular system, which carries the amine to the proliferation zones associated with Clusters 9 and 10. Serotonin in these regions may accelerate the cell cycle progress of neural precursors, increasing the rate of cells entering S phase, and thus the numbers of cells labeled with BrdU (Sandeman et al., 2009). The interpretation of the decrease in BrdU labeling following hyperpolarization of the DGN is less apparent. However, the perfusate in these brain preparations contained detectable levels of serotonin even when there was no DGN stimulation, perhaps suggesting an ongoing release of serotonin at very low levels. Hyperpolarization of the DGN might therefore reduce this release, leading to decreases in cell proliferation. These studies therefore suggest that one role of the DGNs in crayfish (and related decapods), is to regulate adult neurogenesis according to the sensory information that is collected and analyzed in the olfactory lobes and further integrated with visual and mechanosensory information in the accessory lobes.

### The influence of serotonin is lineage-dependent

Serotonin (10^−9^ M) does not alter the rate of BrdU incorporation into neural precursors in the neurogenic niche or proximal migratory streams of *P. clarkii*, but does increase the rate of BrdU uptake into cells in Clusters 9 and 10 where final divisions and differentiation occur. These data indicate that the cell cycle of the 1st- and early 2nd-generation neural precursors are not sensitive to this monoamine. However, the total number of cells composing the niche increases, suggesting that this group of cells is expanding due to the addition of cells from outside the niche, rather than by proliferation of the resident cell population (Benton et al., 2011; Zhang et al., 2011).

Serotonin receptors (5-HT_1α_ and 5-HT_2β_) that are homologous to mammalian subtypes 1A and 2B have been identified and cloned from several crustacean species, including *P. clarkii*, and antibodies raised against conserved regions of the orthologous molecules (Clark et al., 2004; Sosa et al., 2004; Spitzer et al., 2008). Because our BrdU incorporation data (Zhang et al., 2011) indicated lineage-dependent influences of serotonin, we tested this possibility by exploiting the spatial separation of the neural precursor cell generations. *In situ* hybridization with antisense riboprobes specific for 5-HT_1α_ and 5-HT_2β_ receptors revealed strong expression of these mRNAs in several brain regions, including cell clusters 9 and 10. Further, antibodies specific for these receptor subtypes do not bind to the 1st-generation neural precursors in the niche or their daughters as they exit into the migratory streams, but do label the 2nd-generation precursors as they approach the proliferation zones of cell clusters 9 and 10. Experiments using the 5-HT_1α_ specific agonist quipazine maleate salt (QMS) increases the number of BrdU-labeled cells in Cluster 10, and the 5-HT_2β_ antagonist methiothepin mesylate salt (MMS) suppresses neurogenesis in this region, suggesting the involvement of these receptor subtypes in serotonin's effects. However, these pharmacological agents do not alter the rate of BrdU incorporation into the 1st-generation precursors in the niche or their 2nd-generation daughters in the streams proximal to the niche. These studies therefore show that serotonin's influence on adult neurogenesis in the crayfish brain is limited to the late 2nd-generation precursors and their descendants. The conclusion that serotonergic effects are exerted directly on specific generations of neural precursors is reinforced by the distribution of 5-HT_1α_ and 5-HT_2β_ mRNAs and proteins in the later generations of the neural precursor lineage. Taken together, these results indicate that serotonin exerts lineage-dependent effects on adult neurogenesis that are mediated by specific receptor subtypes (Zhang et al., 2011).

### Serotonin promotes the attraction of circulating neural precursors to the niche

**Figure 8:**
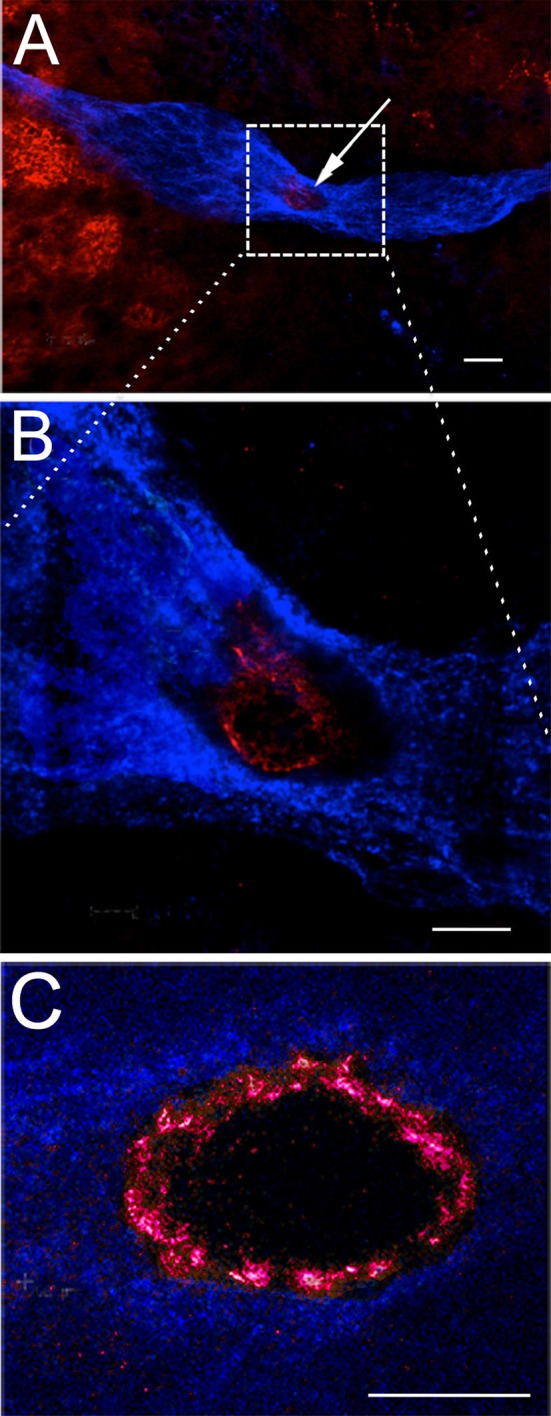
Serotonin is localized in the rim of the vascular cavity. The neurogenic niche in *P. clarkii*, labeled immunocytochemically for glutamine synthetase (blue) and serotonin (red). The vascular cavity is outlined by a rim of serotonin labeling, most clearly seen in **(C)**. The additional serotonin labeling observed in **(A)** to the left of the niche is localized in neural terminals in the accessory lobe. **(A,B)** were contributed by V. Quinan. Scale bars: **(A)**, 20 μm; **(B)**, 10 μm; **(C)**, 10 μm.

**Figure 9:**
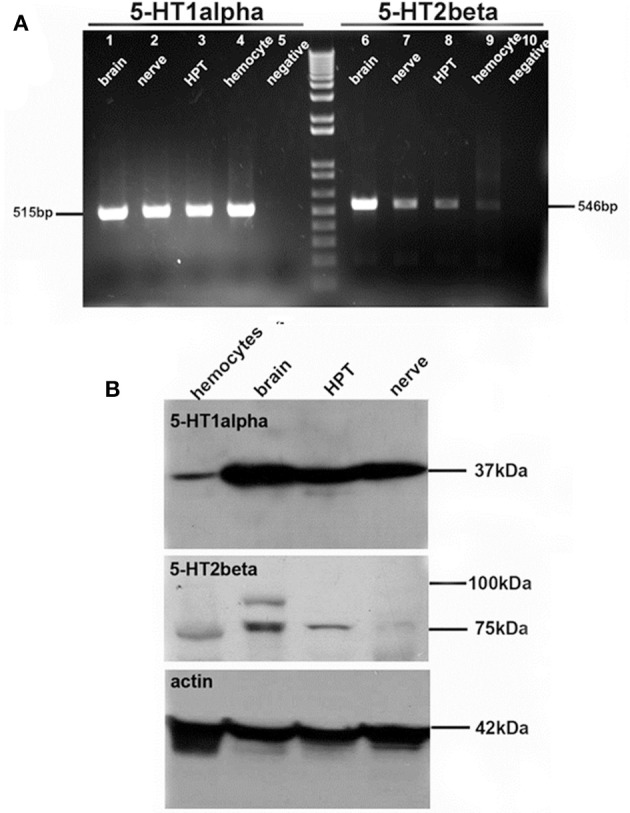
Serotonin receptor subtypes are expressed in hemocytes and brain. **(A)** RT-PCR reveals the expression of 5-HT1α (left) and 5-HT2β (right) receptor subtype mRNAs in brain (lanes 1, 6), nerve (lanes 2, 7), hematopoietic tissue (HPT, lanes 3, 8), hemocytes (lanes 4, 9) and the negative control (lanes 5, 10). **(B)** Western blot for 5-HT1α (top) and 5-HT2β (middle) receptor proteins, with actin control (bottom). Adapted from Benton et al. (2011).

The adoptive transfer experiments described above suggest that the attraction between hemocytes and the niche is highly selective. What is the nature of this attraction? The first clue came from the co-culture experiments in which brains and their associated niches were incubated for 6 h with cells that had been dissociated from several tissue types. As discussed above, only hemocytes showed a significant attraction for the niche *in vitro* (Benton et al., 2011). These studies also probed the basis for this affinity, and showed that hemocyte attraction was severely reduced by the crustacean 5-HT_2β_ receptor antagonist methiothepin mesylate salt (MMS; 10^−8^ M) or by treating crayfish with parachlorophenylalanine (PCPA; an inhibitor of serotonin synthesis) prior to dissecting brains for co-cultures. Although somewhat counter-intuitive, the attraction of hemocytes for the niche also was eliminated if serotonin (10^−9^ M) was introduced into the culture medium, suggesting that the presence of serotonin abolished the natural affinity of hemocytes for the niche. Our interpretation of this result is that serotonin added to the culture medium may destroy or mask a serotonergic signaling gradient associated with the niche (Benton et al., 2011). Finally, serotonin immunoreactivity is localized in the rim of the vascular cavity (the “crown”; Figure 8) in the niche, and hemocytes strongly express the crustacean 5-HT_1α_, as well as 5-HT_2β_ receptors, albeit more weakly (Figure 9) (Benton et al., 2011). Based on these several lines of evidence, we have concluded that serotonin is involved in attracting hemocytes to the niche, building on an extensive history of serotonin as a chemoattractant in the nervous system [reviewed in (Daubert and Condron, 2010)] and the vascular system (Duerschmied et al., 2013; Kang et al., 2013).

The origin of serotonin labeling in the “crown” is still unknown. None of the four cell types in the niche (Chaves da Silva et al., 2012) labels immunocytochemically for serotonin or its rate-limiting enzyme, tryptophan hydroxylase; the niche cells are thus are not considered “serotonergic.” However, no innervation of the niche has yet been discovered that could explain this labeling. We are currently testing the hypothesis that the serotonin immunoreactivity that encircles the vascular cavity results from “borrowed transmitter” taken up through serotonin transporters from the hemolymph and into the terminals of type 1 niche cells. This hypothetical mechanism could provide a means by which serotonin released into the circulation by the DGN could directly influence serotonin levels in the “crown,” and thereby alter the attraction of hemocytes for the niche.

## Future directions

### Next-generation sequencing

In order to characterize the cellular lineage that produces adult-born neurons, we have generated transcriptome data for *P. clarkii* tissues by next-generation sequencing. Genes and members of gene families that are known to play key roles in arthropod neurogenesis and neural and glial differentiation have been identified in the transcriptome. Among these sequences are *SoxB, Achaete-Scute-Complex* and *Snail* transcription factors, *Prospero, Elav* and *Repo. In situ* hybridization for these mRNAs is being combined with *in vivo* cell proliferation studies, to examine the distribution of gene expression for these markers in both the embryonic and adult nervous systems (Brenneis et al., 2017). Further, these probes will be used to examine the differentiation of adoptively transferred hemocytes, to determine whether the lineage of cells produced by hemocytes expresses markers comparable to the natural neurogenic lineage in crayfish. The molecular differentiation of neural precursors and neurons generated in the adult brain will be compared with the spatiotemporal progression of these markers during embryonic neurogenesis. Our ultimate goal is to unravel the genetic network governing the proposed neural differentiation of hemocytes in the procambarid brain.

### Serotonin: a link between the immune and nervous systems?

Studies in the lab of Irene Söderhäll (Uppsala University, Sweden) and by us (Figures 5C,D) have shown that the expression of AST1, the crustacean cytokine that promotes the differentiation and release of semi-granular hemocytes from hematopoietic tissues (Lin et al., 2010, 2011; Cockey et al., 2015), is regulated by serotonin. This association between serotonin and AST1 may suggest a coordinated regulation of the immune and nervous systems, such that hemocyte release is controlled, at least in part, by an agent synthesized and released by the nervous system. Serotonin has potent influences on the rate of neurogenesis, and this may speak not only to its direct effect on the cell cycle, but also to controlling access to neural precursors. For these reasons, we are exploring the connections between serotonin, astakine and adult neurogenesis, to learn how the immune and nervous systems may be influenced by neural agents that alter immune function and by immune agents that alter neurogenesis.

**Figure 10:**
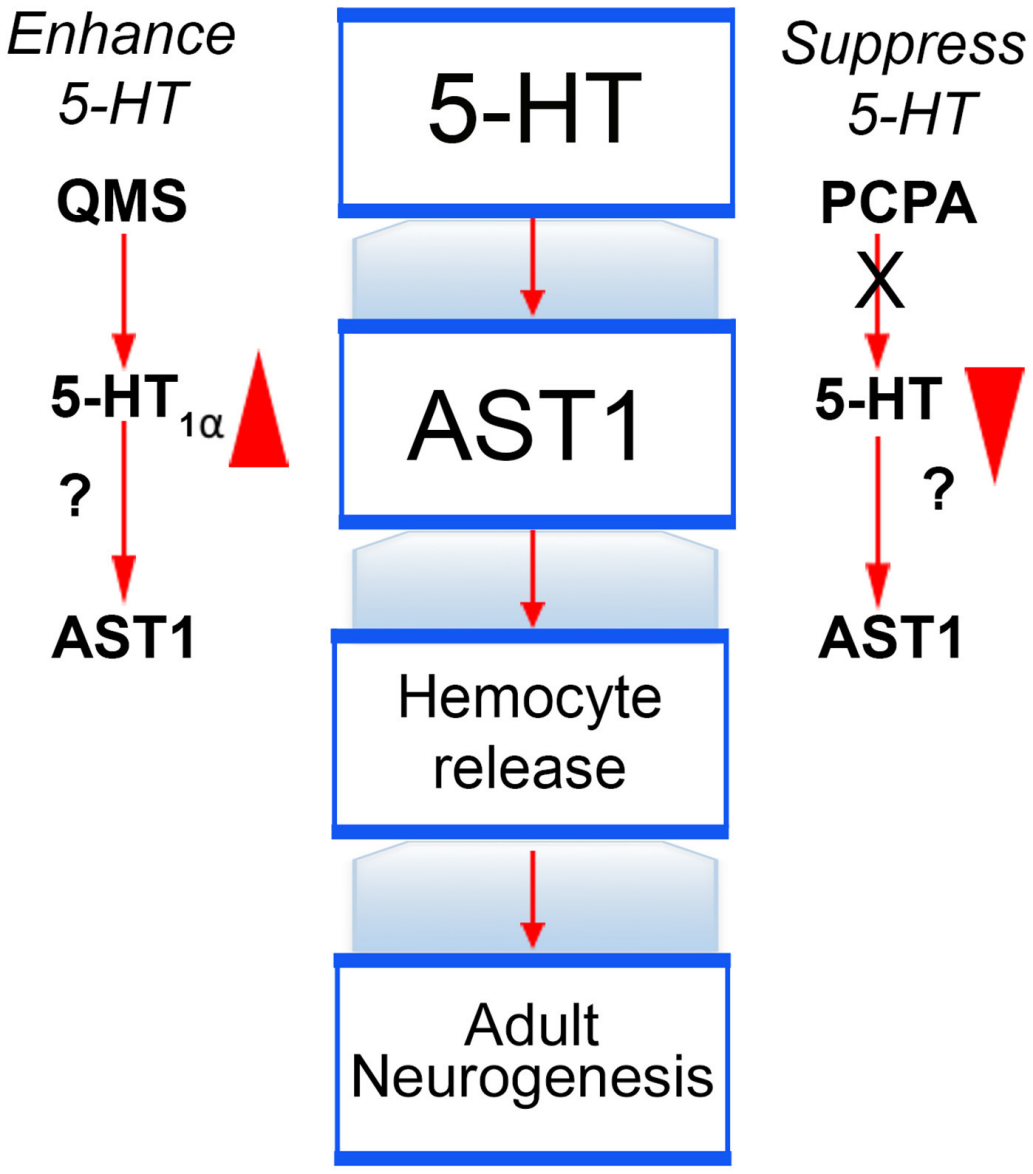
Testing serotonin as a link between the nervous and immune systems. Our working model describing serotonin's role in adult neurogenesis includes stimulating the expression of AST1, which in turn promotes the release of semi-granular hemocytes and accelerates the rate of adult neurogenesis. These relationships will be further tested using agents that enhance or suppress serotonin's action (e.g., quizapine maleate salt [QMS] and PCPA, respectively). While the influence of these compounds on adult neurogenesis in crayfish is known, it has not yet been determined if these effects are mediated by AST1, or result from direct influences of serotonin.

The linkage between serotonin, AST1, hemocyte release and adult neurogenesis will be further explored by, for example, manipulating serotonergic activity with the 5-HT_1α_ specific agonist QMS (Figure 10, left) or depressing the serotonin pathway using the tryptophan hydroxylase inhibitor PCPA (Figure 10, right). Studies using these agents were described above and have been published (Zhang et al., 2011), but their influence on AST1 has not yet been explored; future experiments will fill this gap in our knowledge. Further, RNA interference (RNAi) has been used successfully in the Söderhäll lab (Lin et al., 2010, 2011) to silence AST1 in hematopoietic tissues *in vivo*, which resulted in low hemocyte counts following injection of lipopolysaccharide (LPS) into crayfish; LPS injection normally causes the release of hemocytes and high hemocyte counts. These studies ultimately led to the discovery of an AST1-dependent molecule, crustacean hematopoietic factor (CHF), which is an anti-apoptotic agent. Thus, AST1 promotes the proliferation, release and survival (via CHF) of semi-granular hemocytes. The sequence for *P. clarkii* AST1 has been identified in our transcriptome data, and confirmed by PCR on *P. clarkii* cDNA and subsequent cloning. Knock-down of AST1 will provide a means of separating direct effects of serotonin on adult neurogenesis from those that are mediated by AST1 expression. Overall, these studies will connect our past work on the serotonergic control of adult neurogenesis in crustaceans with cytokine regulation of these processes.

Finally, to better understand the relationship between the immune system and adult neurogenesis, we also are testing whether cells released from immune tissues can be biased toward a neural fate by treatment with a variety of agents, including serotonin, astakine and homogenates of various brain regions. To begin, we have asked whether these agents increase the attraction of cells to the neurogenic niche *in vitro*. Preliminary studies have shown that cultured immune cells treated with homogenate made from the accessory lobes in the brain adopted a more differentiated morphology and also had an enhanced attraction to the niche *in vitro* (Benton et al., 2017). Using adoptive transfer methods, future experiments will examine whether immune cells biased by accessory lobe homogenate are capable of progressing through the neural precursor lineage and whether they express appropriate markers of neural differentiation, finally producing mature neurons. If so, the active agent(s) in the accessory lobe homogenate that promotes the neural biasing of immune cells will be explored and identified.

## Conclusions

The studies reviewed here indicate that the immune and nervous systems work together to generate adult-born neurons in the crustacean brain. Serotonin is one agent that has a potent influence over adult neurogenesis by promoting proliferation in the neural precursor lineage. Serotonin may also serve as a pivotal link between the nervous and immune systems by influencing AST1 expression and by attracting hemocytes to the neurogenic niche. Our future studies will further define the connections between the immune system and adult neurogenesis, and will explore the role that serotonin plays in mediating this relationship.

## Author contributions

BB composed the first draft of this review, which was read critically and edited by JB.

### Conflict of interest statement

The authors declare that the research was conducted in the absence of any commercial or financial relationships that could be construed as a potential conflict of interest.
